# Sexual harassment at German medical schools – a national cross-sectional study

**DOI:** 10.1186/s12909-026-08890-9

**Published:** 2026-02-27

**Authors:** Michelle Förstel, Maximilian Vogt, Sabine Drossard

**Affiliations:** 1 Present Address: Department of Gynecology and Obstetrics, University Hospital Heidelberg, Heidelberg, Germany; 2 German Medical Students’ Association (Bundesvertretung der Medizinstudierenden in Deutschland, bvmd e.V.), Berlin, Germany; 3 Department of Neurosurgery, University Medical Center Greifswald, Greifswald, Germany; 4 Department of General, Visceral, Transplant, Vascular and Pediatric Surgery, University Hospital Würzburg, Würzburg, Germany

**Keywords:** Student Mistreatment, Gender-Based Discrimination, Misconduct, Hierarchy, Medical Education, Medical Studies, Surgery, Career Choice

## Abstract

**Purpose:**

Sexual harassment in medical education is a relevant and well-documented issue that adversely affects students’ mental health, academic engagement and professional development. While several single-faculty studies in Germany have already demonstrated its prevalence, nationally comparable data on the circumstances and consequences of sexual harassment have been lacking. This study provides nationwide data on the extent of sexual harassment, its contextual factors, and consequences for medical students.

**Method:**

A cross-sectional online survey was distributed via the German Medical Students’ Association (bvmd) to medical students across Germany. The questionnaire provided a definition of sexual harassment based on the German General Act on Equal Treatment, illustrated with examples and covered eight thematic areas, addressing both personal experiences and witnessed incidents. Data were analyzed using descriptive statistics, chi-square tests, and logistic regression models.

**Results:**

A total of 5,681 students from 44 German Medical Schools participated. 42,1% reported experiencing sexual harassment, with an increase from preclinical years (29.8%) to clinical years (46.8%) and the practical year (66%). Most affected students reported experiencing repeated harassment. Among final-year students, 73.5% of women and 29.3% of men reported experiencing sexual harassment at some point during their studies. The most common perpetrators were patients (93.8%), fellow students (69.2%) and senior physicians (64.1%). Surgical disciplines exhibited the highest rates of harassment (64.9%), and nearly half (46.0%) of affected students reported that harassment influenced their specialty choice. Harassment was primarily reported in the context of nursing internships, elective placements, the practical year, and extracurricular events. Female students indicated that the perpetrators were almost exclusively male, while male students reported a more heterogeneous distribution. Female students also reported significantly greater distress, largely corresponding to their higher frequency of harassment experiences.

**Conclusion:**

Sexual harassment affects a substantial proportion of medical students in Germany and has far-reaching consequences. These findings highlight the need to strengthen prevention measures and targeted interventions to create a safe and respectful learning environment.

**Supplementary Information:**

The online version contains supplementary material available at https://doi.org/10.1186/s12909-026-08890-9.

## Introduction

Sexual harassment (SH) constitutes a distinct form of power abuse and includes physical, verbal, and non-verbal actions [1]. It both stems from and reinforces existing inequalities, arising from imbalanced power dynamics and simultaneously serving to maintain them [2, 3].

The impact of SH on mental health is extensive and well-documented. Affected individuals exhibit a heightened risk of depression, anxiety disorders, substance abuse, suicidal ideation and overall poorer mental health [4–6]. Additionally, SH is associated with physical health consequences, including hypertension, poorer sleep [6] and chronic back pain [7]. Previous research has demonstrated that medical students and other medical professionals experience a substantial burden due to SH [5, 8, 9]. Furthermore, such experiences can significantly impair academic participation [9].

SH is prevalent across various societal domains, including universities. A 2012 Europewide survey on SH in academic settings among 12,663 students from 16 German universities reported that 19.2% of female students experienced SH during their studies [10].

In medical education, studies indicate an even higher prevalence of SH and gender-based discrimination (GDB): a 2011 meta-analysis of 51 studies including 38,353 trainees reported that 59% of medical students worldwide had experienced at least one form of harassment or discrimination during their studies. Specifically, 33% reported experiencing SH, while 54% reported GBD [11]. At a Swiss Medical School, 16% of 1,033 participants reported sexism or SH in 2023 [5]. There are further reports of SH at medical schools from the U.S [9]. Canada [12], the U.K [13, 14]. and France [15, 16]. Several single-institution studies in Germany support these findings: Studies at the University of Münster, Hannover Medical School, Charité Berlin and the University Hospital Greifswald reported that 23.6% to 58.9% of medical students experienced SH during their education [17–20].

However, a uniform nationwide assessment across all faculties using a standardized instrument has so far been lacking. Data on the experiences of male students remain particularly limited.

Medical education in Germany follows a standardized curriculum set by the Medical Licensure Act (*Approbationsordnung für Ärzte*,* ÄAppO* [21]), which defines duration, content, and examination requirements. It is divided into three phases: (1) Preclinical Science (*Vorklinik)*, 2 years, focusing on basic sciences, followed by the First Medical Examination. (2) Clinical Science (*Klinik)*, 3 years, covering clinical subjects, followed by the Second Medical Examination and (3) Practical Year (PY, *Praktisches Jahr*), a one-year full-time clinical clerkship in certified teaching hospitals, divided into three rotation periods in internal medicine, surgery and an elective clinical discipline. It is followed by the Third Medical Examination.

As part of Preclinical Science, students are required to complete three months of a nursing internship (*Pflegepraktikum)* to familiarize them with fundamental patient care and hospital workflows. It must be completed before the First Medical Examination; some students start it before enrolling in medical school. During Clinical Science, students are required to complete four months of mandatory clinical internship (*Famulatur)*, which offers flexibility in specialty, hospital, or clinic selection, enabling students to explore different medical fields.

In 2024, Germany had 40 state-recognized medical faculties (38 public, 2 private) across 41 locations, offering medical programs accredited under the Medical Licensure Act (*ÄApprO*). Additionally, a growing number of private medical schools offer international qualifications.

This study aims to provide nationwide data on prevalence, circumstances, and impact of SH in medical education in Germany to identify high-risk settings and inform targeted prevention strategies.

## Methods

The questionnaire was developed through an iterative process, incorporating existing questionnaires and theoretical frameworks, with Schoenefeld et al.‘s instrument [17] serving as a reference. To assess completion time and identify potential ambiguities, the questionnaire was piloted on a group of twelve medical students. Based on their feedback, unclear wording was revised, and response options were refined for greater precision.

The final questionnaire (Appendix 2 and 3) comprises eight thematic sections and examines both personal experiences and witnessed instances of SH in medical education. Before starting the survey, participants were provided with a definition of sexual harassment based on the German General Act on Equal Treatment (*Allgemeines Gleichbehandlungsgesetz*, AGG), illustrated by several concrete examples. The survey then includes items on the type of harassment (verbal, non-verbal, physical and digital, 5-point Likert scale), the location of incidents (multiple-choice format), and the role of perpetrators (12 items, 5-point Likert scale). Additionally, it assesses the frequency of harassment across different study phases (8 items, 5-point Likert scale), the medical disciplines in which harassment occurred (multiple-choice format), and the gender of perpetrators (using a numerical ranking scale ranging from 0 = female to 100 = male). Further sections evaluate subjective distress, measured on a 0–10 numerical scale, as well as the impact of SH on academic progression through multiple-choice responses. The questionnaire also includes items on the use of and satisfaction with existing support services, assessed through a 5-point Likert scale and multiple-choice options. Finally, demographic data were collected, including gender, age, prior education, study location, and academic stage (Appendix 2 and 3).

This cross-sectional study was conducted as an online survey between June and September 2024, using LimeSurvey 3 software. The German Medical Students’ Association (*Bundesvertretung der Medizinstudierenden in Deutschland e.V.*,* bvmd)* facilitated survey distribution, which was carried out through deans’ offices, student councils, and social media channels. To prevent multiple submissions, one response per device was allowed via IP-based tracking. The study population included all medical students currently enrolled in medical programs in Germany at the time of data collection. Respondents not actively enrolled in medical training in Germany were excluded from data analysis.

During data collection, it became evident that participants who had not experienced SH often did not complete the questionnaire in full. After the first 1,200 responses, the survey was therefore modified: non-affected participants were only presented with demographic questions and items specifically relevant to them, while items on the circumstances of SH were omitted. The initial 1,200 responses remained included in the analysis; for non-affected participants in this group, missing data were treated as such.

Data analysis was conducted using Python (version 3.11.8) in Visual Studio Code (version 1.87.1). Subgroup sizes were determined separately based on the voluntary provision of demographic data. Incomplete surveys were retained for analyses whenever relevant data were available. To assess differences between two subgroups, the Mann-Whitney U test was applied with a significance level set at α = 0.05, as the data were not normally distributed according to the Shapiro–Wilk test. Box plots illustrate the first and third quartiles, while the median is marked within the box. Differences between groups in categorical variables were analyzed using a chi-square test of independence (χ² (degrees of freedom), p, Cramér’s V). For comparisons between two dichotomous categorical variables, the odds ratio (OR) and confidence intervals (CI) were calculated, with statistical significance determined using Fisher’s exact test (p-value). Associations related to the subjective burden of SH were analyzed using multiple linear regression, with subjective burden assessed on a 0–10 numerical scale, as used in the questionnaire. Analyses were restricted to respondents identifying as male or female with complete data on covariates (*n* = 2797). Dummy coding with predefined reference categories was applied (gender = male, age = 20–24 years, Frequency of SH = never, previous education = none, stage of study = preclinical stage).

## Results

A total of 5,698 medical students participated in the study. After excluding respondents who did not meet the inclusion criteria, 5,681 responses were analyzed, with complete datasets available for 4,158 students (Table 1). Among the respondents, 45.0% had not completed any prior training or studies. Participants were drawn from 44 different locations, including all 41 state-recognized medical schools and three private institutions, with a median of 83 students per location (range: 1 to 276 students) (Appendix 1).

**Table 1:** Cohort description of participants with completed questionnaires

	Preclinical Science (2 years)	Clinical Science (3 years)	Practical Year (1 year)	Total
*n* (full dataset)	1,245 (29,9%)	2,296 (55,2%)	617 (14,8%)	4,158
Gender				
Female	924 (74.2%)	1,799 (78.3%)	512 (83.0%)	3,235 (77,8%)
Male	291 (23.4%)	453 (19.7%)	92 (15.0%)	836 (20,1%)
Other	16 (1.3%)	25 (1.1%)	10 (1.6%)	51 (1,2%)
Prefer not to say	14 (1.1%)	19 (0.9%)	3 (0.4%)	36 (0,9%)
Age				
≤ 19	207 (16.6%)	7 (0.3%)	0 (0%)	214 (5,1%)
20–24	862 (69.3%)	154 (6.8%)	108 (16.8%)	1,124 (27,0%)
25–29	142 (11.5%)	1,455 (63.5%)	428 (69.4%)	2025 (48,7%)
30–34	25 (2.0%)	653 (28.4%)	72 (11.7%)	750 (18,0%)
≥ 35	7 (0.5%)	21 (0.8%)	12 (2.0%)	40 (1,0%)
Prefer not to say	1 (0.1%)	4 (0.1%)	1 (0.1%)	6 (0,1%)

Overall, 42.1% of participants reported experiencing SH during medical school. Additionally, 49.3% stated that they had witnessed SH, while approximately 15% were uncertain whether they had been affected (*n* = 5,681). Gender-specific differences were observed (Table 2), with women experiencing SH significantly more often than men (OR = 8.3 [6.8–10.1], *p* < 0.001) and reporting higher rates of witnessing SH (OR = 3.1 [2.6–3.7], *p* < 0.001). Cases of SH were reported at all study locations with more than five participants.

**Table 2:** Distribution of observed and experienced sexual harassment across genders

	Experienced			Observed		
	Yes	No	Uncertain	Yes	No	Uncertain
Total (*N* = 5,681)	2,392 (42.1%)	2,409 (42.4%)	880 (15.5%)	2,805 (49.3%)	2,008 (35.3%)	868 (15.3%)
Female (*n* = 3,324)	1,760 (52.9%)	1,039 (31.3%)	525 (15.8%)	1,858 (55.9%)	1005 (30.2%)	461 (13.9%)
Male (*n* = 849)	135 (15.9%)	660 (77.7%)	54 (6.4%)	282 (33.2%)	477 (56.2%)	90 (10.6%)
Other (*n* = 52)	27 (51.9%)	18 (34.6%)	7 (13.5%)	30 (57.7%)	14 (26.9%)	8 (15.4%)
Incomplete information (*n* = 1,456)	470 (32.3%)	692 (43.2%)	285 (19.5%)	635 (43.6%)	512 (35.2%)	309 (21.2%)

The proportion of students reporting SH increased significantly across different phases of medical education, rising from 30.0% in Preclinical Science to 47.0% in Clinical Science and reaching 66.4% in the PY (χ² (6) = 276.9, *p* < 0.001, Cramér’s V = 0.14, *n* = 4,158). Among PY students, 73.5% of women and 29.3% of men reported experiencing SH at some point during their studies (OR = 10.3 [6.2–17.4], *p* < 0.001, *n* = 604).

A similar trend was observed for witnessed SH, with reports increasing from 38.0% in Preclinical Science to 52.6% in Clinical Science, and 68.5% in the PY. Among PY students, 72.2% of women and 50.0% of men reported having witnessed SH at least once during their medical training.

Most affected students reported experiencing repeated SH. Among them (*n* = 2,392), 45.3% stated that they experienced harassment one to three times per year, while 50% reported being harassed more than three times per year. Only 5.8% of female and 19.3% of male victims indicated that they had experienced SH only once. Furthermore, 10% of affected individuals reported experiencing SH at least weekly or more frequently. Among PY students, 1% experienced SH at least once a week, while 0.7% reported being harassed several times per day. These findings indicate a significant association between harassment frequency and study phase (χ² (7) = 557.6, *p* < 0.001, Cramér’s V = 0.29).

**Fig. 1:**
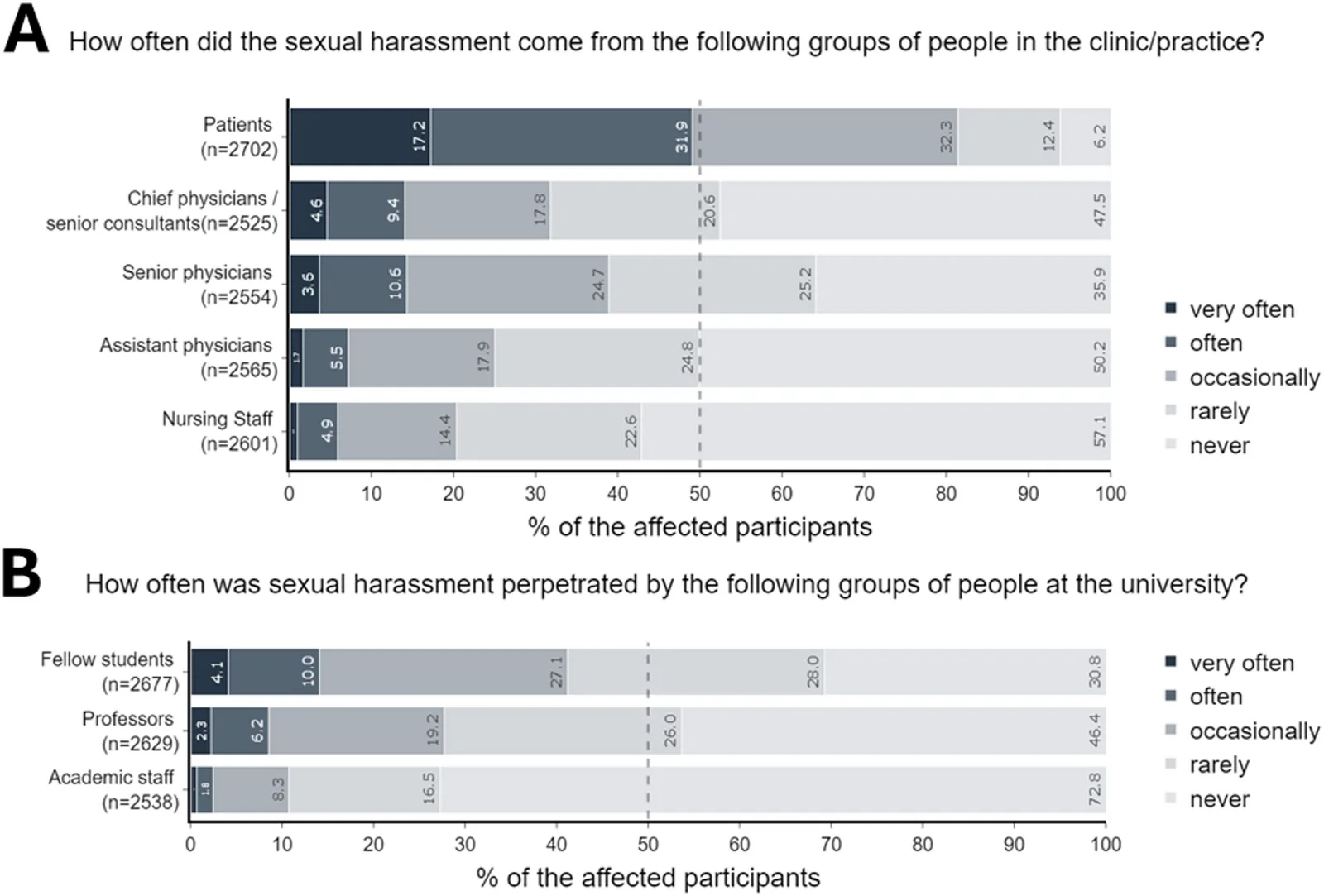
Stacked bar chart depicting the perpetrators of sexual harassment. The professional roles of individuals responsible for initiating sexual harassment are visualized in a stacked bar chart. The frequency of reported harassment is color-coded, ranging from dark (frequent occurrences) to light (never experienced). Only responses from students who had prior interactions with these groups and had either experienced or observed sexual harassment were included in the analysis. The chart differentiates between two categories of perpetrators: (**A**) individuals from clinical or practice settings and (**B**) individuals from academic institutions (universities)

The most frequently reported form of SH was verbal harassment (*unwanted*,* sexually connoted verbal expression*), experienced by 97.7% of affected individuals (*n* = 2,392), with 33.4% reporting it often or very often. This was followed by non-verbal harassment (*unwanted*,* sexualized actions*,* looks or gestures*), reported by 84.3% (16.9% often or very often). Physical harassment (*unwanted physical touching or advances*) was reported by 62.9% (4.3% often or very often), while digital harassment (*unwanted behavior with a sexual connotation via electronic means of communication*) was the least common, affecting 26.9% of respondents (4% often or very often). The most common perpetrators of harassment were patients, followed by fellow students, as well as specialist and senior physicians (Fig. 1).

**Fig. 2:**
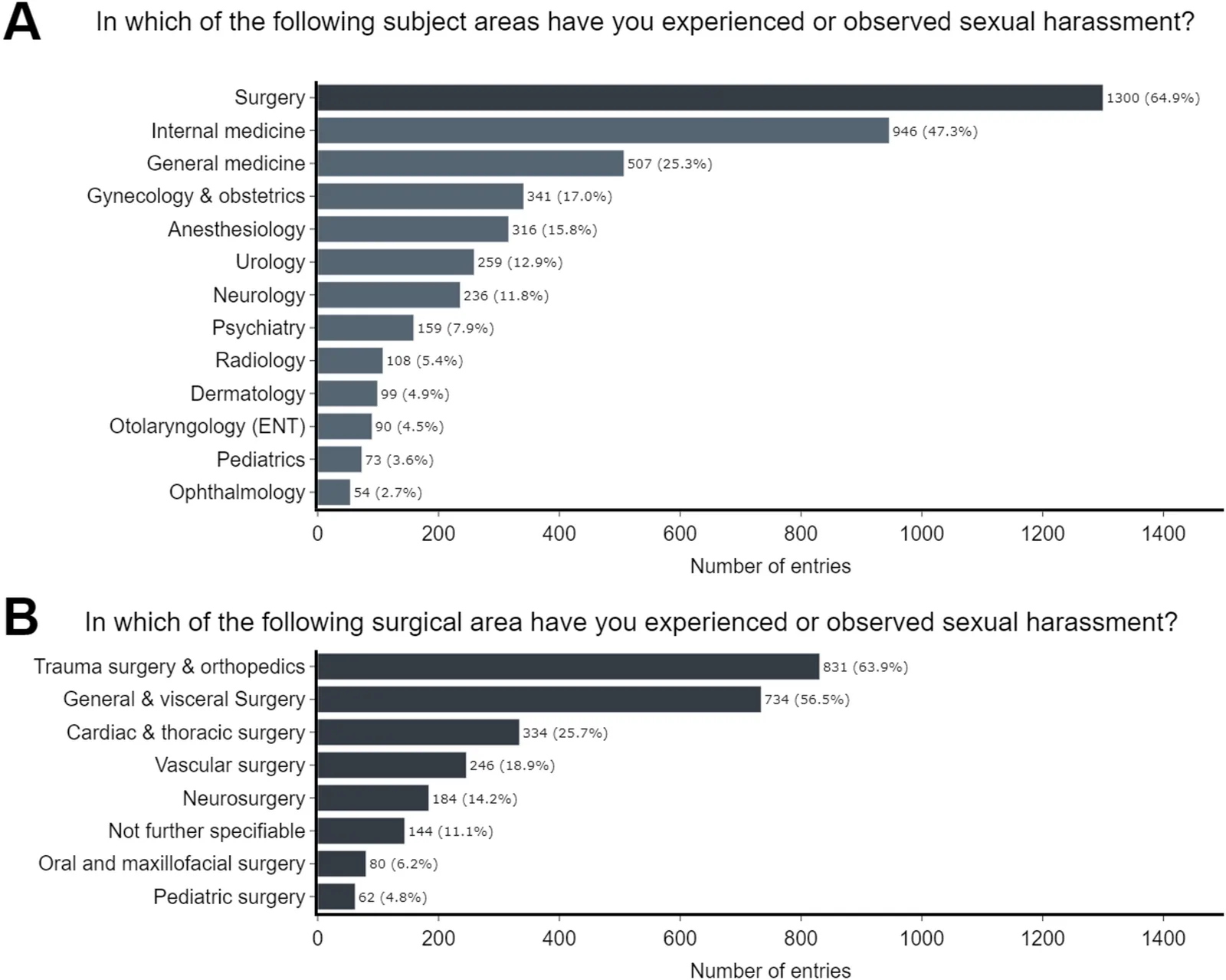
Bar chart on sexual harassment by specialty. **A** Bar chart displaying the absolute frequencies of reported sexual harassment by specialty (multiple responses possible, *n* = 2,399). **B** Bar chart focusing on surgical specialties, showing absolute frequencies of reported sexual harassment (multiple responses possible, *n* = 1,304)

**Fig. 3:**
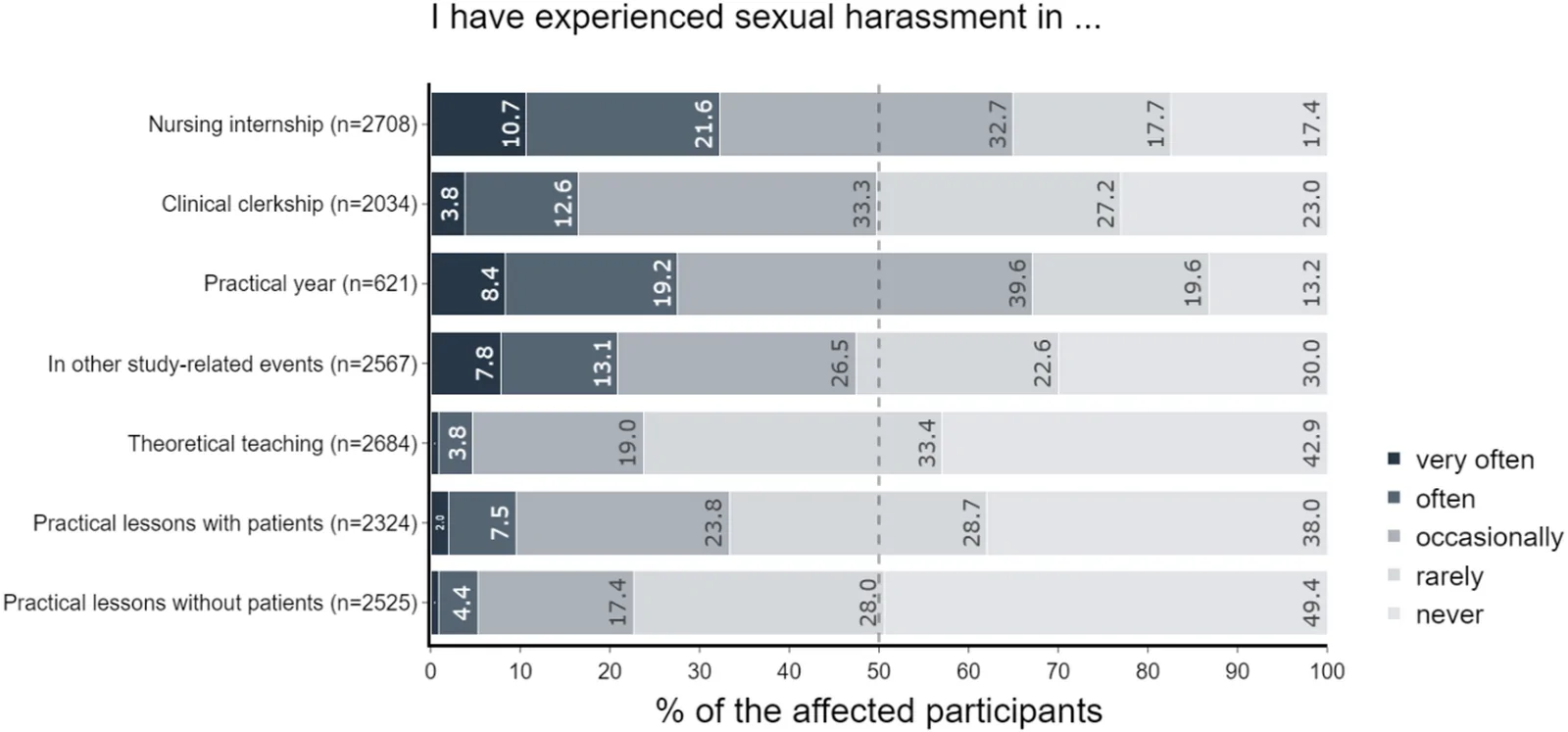
Stacked bar chart of study-related courses where sexual harassment occurred. The frequency of incidents is represented using a color gradient, with darker shades indicating more frequent occurrences. The analysis includes only responses from students who were already enrolled in these courses and had either personally experienced or witnessed sexual harassment

Affected individuals reported experiencing SH most frequently in the field of surgery, particularly in trauma surgery/orthopedics and general and visceral surgery. Additionally, SH was reported to occur more frequently in internal medicine and general practice (Fig. 2). SH was primarily reported in the context of nursing internships, elective placements, the PY, and extracurricular events (Fig. 3). Female students indicated that the perpetrators were almost exclusively male, with a median rating of 100% on a scale from 0% (female only) to 100% (male only) (IQR = 3%, *n* = 1,746), while male students reported a more heterogeneous distribution, with a median rating of 67% (IQR = 90%, *n* = 130).

SH was reported most frequently in university hospitals (73.6%), followed by teaching hospitals (53.4%) and university buildings such as libraries and lecture halls (36.9%). Additionally, 32.7% of respondents experienced harassment in non-teaching hospitals, while 25.9% reported incidents occurring in medical practices (*n* = 2,001).

**Fig. 4:**
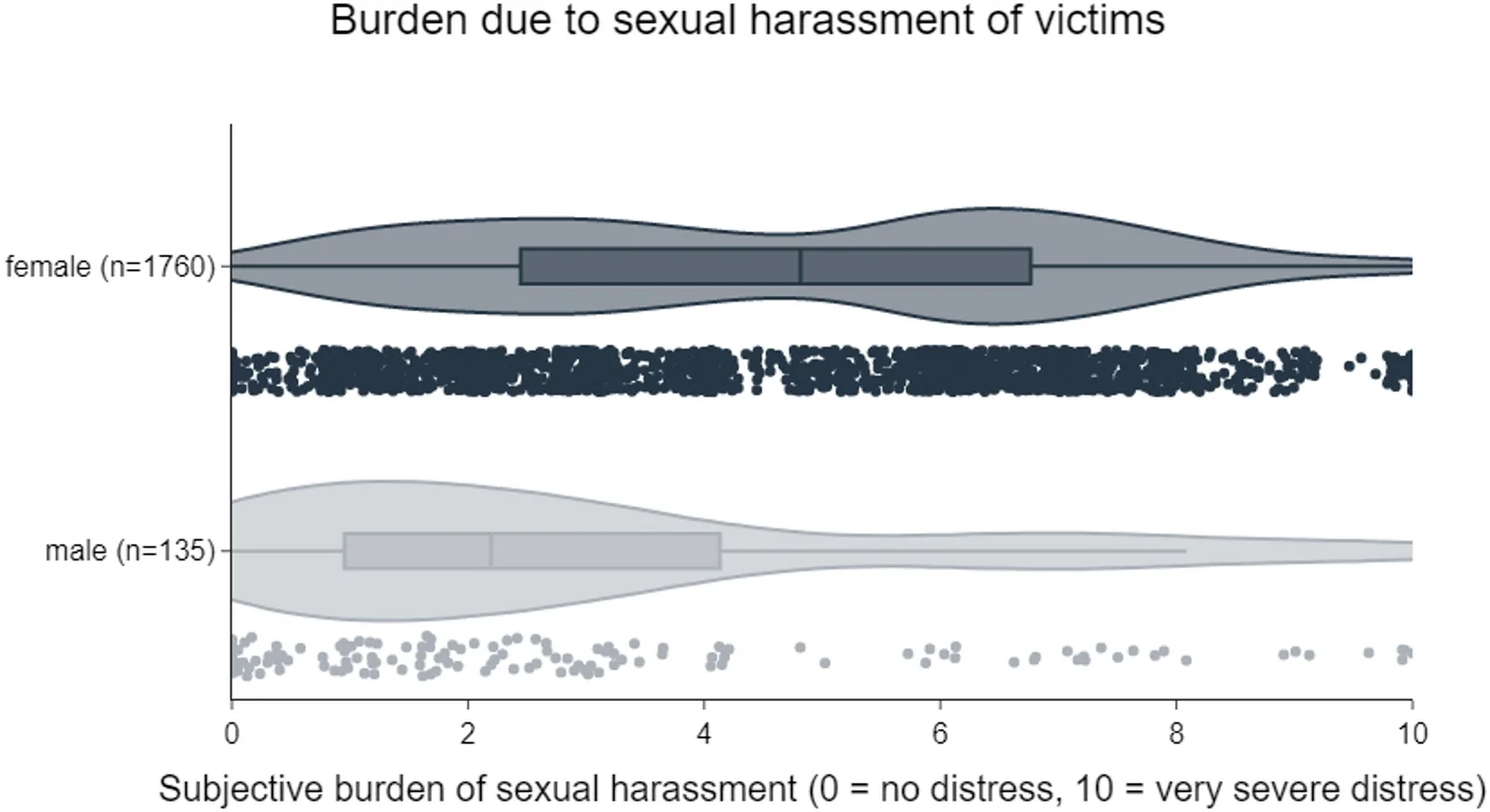
Sexual harassment burden on victims represented as a violin plot. The subjective burden of sexual harassment was assessed using a 0–10 scale and visualized as a violin plot, separated by gender (male and female). Individual responses are represented as points, with a jitter of 0.25 applied to enhance visibility

The subjective burden of SH, assessed on a 0–10 scale (0 = no distress, 10 = very severe distress), varied among affected individuals. Female victims reported significantly higher distress levels (median = 5.0, IQR = 4.5) compared to male victims (median = 2.0, IQR = 3, *p* < 0.001) (Fig. 4).

Linear regression analysis of perceived burden indicates that distress is primarily influenced by the frequency of SH, with women experiencing harassment significantly more often. Gender, prior education, and curricular stage play a minor role in perceived distress. While female gender is associated with a slightly increased level of distress, individuals who had already completed medical training reported a lower perceived burden (Table 3).

**Table 3:** Regression analysis of the subjective burden of sexual harassment. This table presents the results of the linear regression analysis examining factors associated with the subjective burden of sexual harassment. The dependent variable is the reported burden on a scale of 0–10

Subjective Burden of Sexual Harassment (0–10 Pts.)	B	SE	t	*p*	β
Constant	0.50	0.15	3.24	< 0.001	-1.06
Gender (Reference = Male)					
Female	0.79	0.13	6.34	< 0.001	0.29
Age (Reference = 20–24)					
< 19	-0.56	0.23	-2.44	0.015	-0.20
25–29	0.35	0.11	3.12	0.002	0.13
30–34	0.37	0.21	1.79	0.073	0.14
> 35	0.12	0.56	0.21	0.833	0.04
Absolute Frequency (Reference = Never)					
Once	0.81	0.17	4.90	< 0.001	0.30
Approx. 1–3 x per year	2.13	0.14	15.30	< 0.001	0.78
Approx. 1–3 x per quarter	3.79	0.16	24.35	< 0.001	1.39
Approx. 1–3 x per month	4.51	0.19	23.80	< 0.001	1.65
Approx. 1–3 x per week	5.67	0.29	19.29	< 0.001	2.08
(Almost) daily	5.24	0.65	8.12	< 0.001	1.92
Several times a day	7.44	1.11	6.72	< 0.001	2.73
Previous Education (Reference = None)					
Yes, training in the medical-related field	-0.34	0.12	-2.83	0.005	-0.13
Yes, in the non-medical field	-0.07	0.15	-0.50	0.615	-0.03
Yes, other activities in medicine-related	-0.08	0.12	-0.66	0.51	-0.03
Stage of Study (Reference = Preclinical Science)					
Clinic Science	-0.10	-0.10	0.11	-0.92	-0.03
Practical Year	-0.29	-0.29	0.16	-1.84	-0.11
R² = 0.359, adjust. R² = 0.355	F = 91.4, F: *p* < 0.001				

Among affected students, only 12.7% reported an incident (*n*=2392). 32.8% of those affected stated that SH had no negative impact on their studies or career, whereas 68.1% actively avoided specific situations or individuals due to their experiences. Furthermore, 52.2% reported that harassment had affected their psychological well-being, while 58.6% indicated that it influenced their career choices, and 46.0% reported an impact on their choice of medical specialty (*n* = 516).

## Discussion

This cross-sectional study adds detail on when, where, and by whom harassment occurs during medical training, thereby providing a more nuanced basis for targeted prevention strategies. With 5,681 participants, it offers sufficient statistical power for subgroup analyses, that were not feasible in earlier work.

Because male students are less frequently affected by sexual harassment, they were excluded from some previous studies [22] or constitute only a very small subgroup (e.g. 19 in Münster [17]), thus preventing meaningful subgroup analyses. Women are more likely than men to label certain behaviors as SH [23]. In our cohort, however, 135 male students reported experiences, allowing us to examine their perspective in greater detail - for example regarding the gender of perpetrators and the perceived burden.

The data suggest that the prevalence of SH in medical education in Germany may be higher than previously assumed, with a concerning increase over the course of medical training. Among PY students, two-thirds reported experiencing SH, with three out of four female PY students affected. The higher prevalence among final-year students is consistent with cumulative exposure to clinical environments and contact groups over time, underscoring the need for early preventive measures during preclinical and early clinical phases. This pattern aligns with prior work indicating a rising prevalence over the course of medical school [5, 24] - a factor that should be considered when interpreting prevalence rates.

### Comparison of prevalence rates with existing German data

Several German studies have documented the prevalence of SH in medical education and academic medicine in the last years: At the Medical School in Hannover, 343 students participated in a survey, with 29.2% reporting personal experiences of SH and 53.9% reporting observations [18]. In Münster, a large study of 623 students found a prevalence of 58.9% for SH [17]. At the Charité Berlin, 964 medical students and 275 teaching staff participated; 23.6% of students and 19.2% of lecturers reported experiences of SH, while 49.6% of students and 32% of lecturers reported discriminatory behavior [19].

Furthermore, several recent German studies reported high rates of SH and GBD: At Greifswald University Hospital, 623 staff and 143 medical students were surveyed, with 44,6% of all participants but 63,7% of students reporting SH, GBD, or violence during their career [20]. Stock et al. surveyed 759 medical students across 31 German medical faculties and found that 65% experienced GBD, which was strongly linked to specialty choice, with affected students less likely to pursue surgical disciplines. Their definition of GDB encompassed SH items as well (e.g. unwanted touch) [25]. Tameling et al. studied 392 women (235 students and 157 physicians) across five university hospitals in northern Germany in 2023 in a qualitative study, reporting GDB, including SH, in almost 75% of participants [22].

Differences between our findings and those previous studies may partly reflect methodological variation (quantitative vs. qualitative), including how sexual harassment is defined, the categories of perpetrators included, and the exact wording of survey items. Notably, some studies examine SH, GBD and violence in combination, while our survey focused specifically on experiences of SH (defined according to the German General Act on Equal Treatment (AGG) and illustrated with examples), without addressing broader issues such as sexism or GDB. Because SH and GBD overlap conceptually and surveys vary in scope, direct numerical comparisons with the studies that report substantially higher overall rates of GDB [20, 22, 25] should be interpreted cautiously and primarily within each study’s definitional frame.

Furthermore, awareness of SH has changed over time [23] and may also differ between faculties, particularly where local initiatives or awareness campaigns have already been implemented. Such differences could partly explain variability in prevalence rates observed across German studies.

Beyond student populations, Clemens et al. (2025) surveyed 1,403 physicians and 2,365 nurses at four university hospitals in southwest Germany, reporting lifetime SH prevalence of 74.2% among female physicians and 51.2% among male physicians [26]. Comparable results were found in a 2021 U.S. survey of 830 faculty members in academic medicine, where 71.9% of women and 44.9% of men reported gender harassment [27]. These findings closely align with the prevalence rates observed among final-year students (73.5% of women, 29.3% of men) in our study.

### Perpetrators

In our survey, 93.8% of affected students reported SH by patients, confirming patients as a key perpetrator group in line with previous studies: Schoenefeld et al. likewise identified patients as the most frequent perpetrators of physical harassment, while 91.7% of affected female and 8.3% of affected male students reported verbal harassment by patients [17]. Ludwig et al. found a lower proportion (26.6% of all students) [19], reflecting that their figure included the entire sample rather than affected students only. Clemens et al. also confirmed patients as key perpetrators among health professionals. These numbers align with international surveys among U.S. and Swiss medical students that report high rates of SH by patients [5, 24]. Patient contact occurs not only in structured clinical courses but also in decentralized settings, such as the nursing internship, where students are frequently exposed to SH. Given that younger students are at a higher risk [9, 10, 28], targeted preventive interventions - including raising awareness of available support and reporting services- should be implemented early in medical training.

Regarding teaching staff, the students in our study reported experiences of SH predominantly in interactions with chief physicians, senior physicians, and specialists, highlighting the impact of hierarchical structures. Anti-harassment training should be implemented into faculty development programs to ensure that educators recognize, prevent, and appropriately respond to incidents of harassment.

Fellow students and other hospital staff have so far received little attention as perpetrators, leaving a gap in understanding the full spectrum of harassment dynamics in medical education. A notable and underexplored finding of this study is the high prevalence of SH by peers, with two-thirds of affected individuals reporting such experiences. While peer harassment has been discussed more prominently in U.S. colleges with residential campus structures [28], it remains underexplored in Germany. As medical students are the future physicians and leaders, such behaviors risk perpetuating hierarchical sexism and undermining workplace culture in the future. Therefore, peer harassment should be explicitly addressed within the preventive frameworks of medical schools. Strategies include early sensitization, integration of gender-awareness and professionalism curricula, clear expectations of peer conduct, and the promotion of positive role models in leadership positions.

### Consequences of SH

Our findings indicate that SH causes not only psychological and emotional distress but also negatively impacts professional development. Subjective distress was strongly correlated with the frequency of SH incidents. Women reported significantly higher levels of stress than men, which is consistent with their eightfold higher exposure to harassment.

Consistent with previous national and international research [15, 25, 29–31], surgical specialties were identified in our study as high-risk environments for harassment. Approximately half of the students reported avoiding certain specialties due to negative experiences. This falls in line with previous research demonstrating that SH and GDB in clinical education can deter students from pursuing specific fields [25, 31–33]. A systematic review further highlighted that GDB particularly discourages female students from entering surgical careers [31], resulting in a substantial loss of expertise and diversity in the medical profession. Female physicians continue to experience harassment during postgraduate training, highlighting the persistent nature of the issue [4, 20, 26, 34]. Given the growing shortage of healthcare professionals, this problem affects not only the individuals concerned but also the healthcare system as a whole. If institutions fail to implement structural reforms, these patterns will persist, limiting workforce diversity and reinforcing power asymmetries.

Beyond individual career choices, SH can also directly impact team communication and the quality of patient care [35]. Many students reported avoiding specific individuals - potentially including patients - due to negative experiences, which could, in turn, affect patient safety.

### Measures and policies

Despite the increasing awareness generated by previous studies and the implementation of individual pilot projects and initiatives [36, 37], SH in medical education remains insufficiently addressed. The persistence of the problem suggests that previous measures have either lacked depth or have not been implemented on a nationwide scale, underscoring the need for comprehensive, systemic interventions.

Workplace structures and institutional tolerance of inappropriate behavior are recognized risk factors for SH [3, 38]. Additionally, the dependency dynamics of clinical training create barriers for students seeking support, further complicating efforts to mitigate GBD and SH [39]. To address this, medical faculties and clinics must foster safe and respectful environments through proactive, evidence-based prevention strategies and support mechanisms that extend beyond formal policies and specifically target high-risk settings and groups.

### Limitations

This study has several limitations. Although prior research suggests that self-selection bias in SH surveys may not substantially affect outcomes [40], both non-response and self-selection bias must be considered in our voluntary survey. Female students were overrepresented in our sample (77.9%) compared to the national average (64.6% in 2023) [41]; particularly in the final-year cohort. This may indicate that students with prior experiences of SH were more likely to participate, which could lead to an overestimation of prevalence. Conversely, underreporting is a well-documented problem in SH research, and it is equally plausible that affected students chose not to participate. The direction of bias is therefore uncertain. Nevertheless, our findings are consistent with previous German and international studies, supporting the validity of the observed patterns.

Due to varying local response rates, faculty-level prevalence rates cannot be interpreted conclusively. However, the multicenter design allows for the identification of common trends across institutions, particularly when focusing on the large subgroup of affected students (2,392 experienced and 2,805 witnessed SH). This enables meaningful analyses of relative patterns such as gender differences, perpetrator groups, and the circumstances in which SH occurs.

Furthermore, exposure to different specialties and teaching environments may influence the likelihood of SH, but this variable was not captured in the present study. Nevertheless, our data highlight the central role of high-risk specialties, especially surgical fields, consistent with previous reports [16, 25, 29, 31, 32, 34].

The survey instrument was developed by the authors, adapted from a prior questionnaire used by Schoenefeld et al. [17]. While it underwent pilot testing, it has not been formally validated.

Despite these limitations, the large sample size, use of a uniform survey instrument, and the national reach with structural variables provide a robust basis for subgroup analyses and to inform targeted prevention strategies. Future research should aim to address potential selection biases and contextual factors by employing longitudinal or mixed-method approaches to achieve a more comprehensive understanding of SH in medical training.

## Conclusion

Our study highlights the high prevalence of SH in German medical education and its significant impact on affected students. Sexual harassment in medical education is more than an individual experience; it is a reflection of entrenched institutional power dynamics. Structural reforms are urgently needed within medical schools and clinical training environments to foster safe and respectful learning and working environments for all students. Effective prevention strategies must go beyond individual-level interventions and be institutionally and culturally embedded to drive long-term systemic change. Addressing SH at a structural level is critical to advance equality and inclusion, as its effects extend beyond medical education, influencing career choices, workplace retention, and overall representation in leadership roles within the medical field.

## Supplementary Information


Supplementary Material 1. Appendix 1: Distribution of feedback per faculty.



Supplementary Material 2. Appendix 2: PDF of the original questionnaire (in German).



Supplementary Material 3. Appendix 3: PDF of translated questionnaire.


## Data Availability

The datasets used and analyzed during the current study are available from the corresponding author on reasonable request.

## References

[1] Federal Anti-Discrimination Agency. Sexual Harassment in the Workplace (Antidiskriminierungsstelle des Bundes. Sexuelle Belästigung am Arbeitsplatz.). www.antidiskriminierungsstelle.de. https://www.antidiskriminierungsstelle.de/DE/ueber-diskriminierung/lebensbereiche/arbeitsleben/sexuelle-belaestigung-am-arbeitsplatz/sexuelle-belaestigung-am-arbeitsplatz-node.html. Accessed 2 Jan 2025.

[2] Täuber S, Loyens K, Oertelt-Prigione S, Kubbe I. Harassment as a consequence and cause of inequality in academia: A narrative review. EClinicalMedicine. 2022;49:101486. 10.1016/j.eclinm.2022.101486. 35747190 PMC9167878

[3] Johnson PA, Widnall SE, Benya FF, editors. Sexual Harassment of Women. Washington, D.C.: National Academies; 2018. 10.17226/24994.

[4] Hu Y-Y, Ellis RJ, Hewitt DB, Yang AD, Cheung EO, Moskowitz JT, et al. Discrimination, Abuse, Harassment, and Burnout in Surgical Residency Training. N Engl J Med. 2019;381:1741–52. 10.1056/nejmsa1903759. 31657887 PMC6907686

[5] Barbier JM, Carrard V, Schwarz J, Berney S, Clair C, Berney A. Exposure of medical students to sexism and sexual harassment and their association with mental health: a cross-sectional study at a Swiss medical school. BMJ Open. 2023;13. 10.1136/bmjopen-2022-069001. PMC10151891 37105707

[6] Thurston RC, Chang Y, Matthews KA, von Känel R, Koenen K. Association of Sexual Harassment and Sexual Assault With Midlife Women’s Mental and Physical Health. JAMA Intern Med. 2019;179:48. 10.1001/jamainternmed.2018.4886. 30285071 PMC6322939

[7] Stock SR, Tissot F. Are there health effects of harassment in the workplace? A gender-sensitive study of the relationships between work and neck pain. Ergonomics. 2012;55:147–59. 10.1080/00140139.2011.598243. 21864223

[8] Mushtaq M, Sultana S, Imtiaz I. The Trauma of Sexual Harassment and its Mental Health Consequences Among Nurses. J Coll Physicians Surg Pak. 2015;25:675–9. 26374365

[9] McClain T, Kammer-Kerwick M, Wood L, Temple JR, Busch-Armendariz N. Sexual Harassment Among Medical Students: Prevalence, Prediction, and Correlated Outcomes. Workplace Health Saf. 2021;69:257–67. 10.1177/2165079920969402. 33331247

[10] List K, Feltel T. Sexuelle Gewalt an Hochschulen. Die Hochschule: Journal für Wissenschaft und Bildung. 2015;24:115–28. 10.25656/01:16225

[11] Fnais N, Soobiah C, Chen MH, Lillie E, Perrier L, Tashkhandi M, et al. Harassment and discrimination in medical training: A systematic review and meta-analysis. Acad Med. 2014;89:817–27. 10.1097/ACM.0000000000000200. 24667512

[12] Phillips SP, Webber J, Imbeau S, Quaife T, Hagan D, Maar M, et al. Sexual Harassment of Canadian Medical Students: A National Survey. EClinicalMedicine. 2019;7:15–20. 10.1016/j.eclinm.2019.01.008. 31193665 PMC6537541

[13] Skan O, Tregidgo L, Tizzard J, Westlake I, Joji N. Examining medical students’ experience of gender-based discrimination and sexual harassment from clinical teachers at a UK medical school. Med Teach. 2025;47. 10.1080/0142159X.2024.2331034. 38564885

[14] Broad J, Matheson M, Verrall F, Taylor AK, Zahra D, Alldridge L, et al. Discrimination, harassment and non-reporting in UK medical education. Med Educ. 2018;52:414–26. 10.1111/medu.13529. 29574959

[15] Duba A, Messiaen M, Boulangeat C, Boucekine M, Bourbon A, Viprey M, et al. Sexual harassment exposure and impaired mental health in medical students. The MESSIAEN national study. J Affect Disord. 2020;274:276–81. 10.1016/J.JAD.2020.05.100. 32469816

[16] Lisan Q, Pigneur B, Pernot S, Flahault C, Lenne F, Friedlander G, et al. Is sexual harassment and psychological abuse among medical students a fatality? A 2-year study in the Paris Descartes School of Medicine. Med Teach. 2021;43:1054–62. 10.1080/0142159X.2021.1910225. 33882785

[17] Schoenefeld E, Marschall B, Paul B, Ahrens H, Sensmeier J, Coles J, et al. Medical education too: sexual harassment within the educational context of medicine – insights of undergraduates. BMC Med Educ. 2021;21:81. 10.1186/s12909-021-02497-y. 33526025 PMC7852293

[18] Jendretzky K, Boll L, Steffens S, Paulmann V. Medical students’ experiences with sexual discrimination and perceptions of equal opportunity: A pilot study in Germany. BMC Med Educ. 2020;20. 10.1186/s12909-020-1952-9. PMC7036258 32087726

[19] Ludwig S, Jenner S, Berger R, Tappert S, Kurmeyer C, Oertelt-Prigione S, et al. Perceptions of lecturers and students regarding discriminatory experiences and sexual harassment in academic medicine – results from a faculty-wide quantitative study. BMC Med Educ. 2024;24. 10.1186/s12909-024-05094-x. PMC11044556 38658938

[20] Buchhold B, Wille J, Stracke S, Lutze S. Prävalenz sexualisierter Belästigung in einem Krankenhaus der Maximalversorgung: Eine Querschnittserhebung [Prevalence of sexualized harassment in an academic hospital - a cross-sectional survey]. MMW Fortschr Med. 2024;166(Suppl 6):9–18. 10.1007/s15006-024-4421-2. 39653950

[21] Licensing Regulations for Physicians, last Changed 07. June 2023 (Approbationsordnung Für Ärzte, in der Änderungsfassung vom 07. June 2023). https://www.gesetze-im-internet.de/_appro_2002/BJNR240500002.html

[22] Tameling J-F, Lohöfener M, Bereznai J, Phuong T, Tran A, Ritter M, et al. Extent and types of gender-based discrimination against female medical students and physicians at five university hospitals in Germany -results of an online survey. GMS J Med Educ. 2023;40(6):Doc66. 10.3205/zma001648. 38125897 PMC10728668

[23] Zhou Y, Nguyen H-HD, Revier MS, Krueger KR, Sackett PR. An updated examination of gender differences in sexual harassment perception: A meta-analysis and a survey study. J Occup Health Psychol. 2024;29:373–408. 10.1037/ocp0000391. 39699626

[24] Mahurin HM, Garrett J, Notaro E, Pascoe V, Stevenson PA, DeNiro KL, et al. Sexual harassment from patient to medical student: a cross-sectional survey. BMC Med Educ. 2022;22. 10.1186/s12909-022-03914-6. PMC9710121 36451194

[25] Stock J, Kaifie A. The effects of gender discrimination on medical students‘ choice of specialty for their (junior) residency – a survey among medical students in Germany. BMC Med Educ. 2024;24. 10.1186/s12909-024-05579-9. PMC11140860 38816875

[26] Clemens V, Kuchenbaur M, Richter C, Oertelt-Prigione S, Taubner S, Fegert JM. Sexual Harassment in Academic Medicine in Germany. JAMA Netw Open. 2025;8:e2518237–2518237. 10.1001/JAMANETWORKOPEN.2025.18237. 40569598 PMC12203272

[27] Jagsi R, Griffith K, Krenz C, Jones RD, Cutter C, Feldman EL, et al. Workplace Harassment, Cyber Incivility, and Climate in Academic Medicine. JAMA. 2023;329:1848. 10.1001/jama.2023.7232. 37278814 PMC10245188

[28] Wood L, Hoefer S, Kammer-Kerwick M, Parra-Cardona JR, Busch-Armendariz N. Sexual Harassment at Institutions of Higher Education: Prevalence, Risk, and Extent. J Interpers Violence. 2021;36:4520–44. 10.1177/0886260518791228. 30071790 PMC10676016

[29] Gianakos AL, Freischlag JA, Mercurio AM, Haring RS, LaPorte DM, Mulcahey MK, et al. Bullying, Discrimination, Harassment, Sexual Harassment, and the Fear of Retaliation During Surgical Residency Training: A Systematic Review. World J Surg. 2022;46:1587–99. 10.1007/s00268-021-06432-6. 35006329

[30] Nora LM, McLaughlin MA, Fosson SE, Stratton TD, Murphy-Spencer A, Fincher R-ME, et al. Gender Discrimination and Sexual Harassment in Medical Education: Perspectives Gained by a 14-school Study. Acad Med. 2002;77:1226–34. 10.1097/00001888-200212000-00018. 12480632

[31] Peel JK, Schlachta CM, Alkhamesi NA. A systematic review of the factors affecting choice of surgery as a career. Can J Surg. 2018;61:58–67. 10.1503/cjs.008217. 29368678 PMC5785290

[32] Skorus U, Karpińska I, Kominko A, Romaniszyn M. Why do Polish medical students resign from pursuing surgical careers? A survey study. Pol J Surg. 2020;92:1–5. 10.5604/01.3001.0013.7955. 32310819

[33] Stratton TD, McLaughlin MA, Witte FM, Fosson SE, Nora LM. Does Students’ Exposure to Gender Discrimination and Sexual Harassment in Medical School Affect Specialty Choice and Residency Program Selection? Acad Med. 2005;80:400–8. 10.1097/00001888-200504000-00020. 15793027

[34] Jariwala K, Wilson CA, Davidson J, Hu J, Symonette C, de Ribaupierre S, et al. A Canadian National Survey Study of Harassment in Surgery—Still a Long Way to Go. J Surg Educ. 2024;81:1075–82. 10.1016/J.JSURG.2024.05.010. 38834433

[35] Hayward L, Mott NM, McKean EL, Dossett LA. Survey of student mistreatment experienced during the core clinical clerkships. Am J Surg. 2023;226:13–8. 10.1016/j.amjsurg.2022.12.022. 36669940

[36] Drossard S, Warnken I. Teaching medical students to navigate workplace harassment – preliminary experiences from a pilot workshop in Germany. BMC Med Educ. 2025;25:1251. 10.1186/s12909-025-07853-w. 40931361 PMC12421763

[37] Hock LE, Barlow PB, Scruggs BA, Oetting TA, Martinez DA, Abràmoff MD, et al. Tools for Responding to Patient-Initiated Verbal Sexual Harassment: A Workshop for Trainees and Faculty. MedEdPORTAL. 2021;17:11096. 10.15766/mep_2374-8265.11096. 33598539 PMC7880260

[38] Willness CR, Steel P, Lee K. A Meta-Analysis of the Antecedents and Consequences of Workplace Sexual Harassment. Pers Psychol. 2007;60:127–62. 10.1111/j.1744-6570.2007.00067.x.

[39] Chan ZC, Chien WT, Henderson S. Power dynamics in the student-teacher relationship in clinical settings. Nurse Educ Today. 2017;49:174–9. 10.1016/j.nedt.2016.11.026. 27984796

[40] Rosenthal M, Freyd J. Sexual Violence on Campus: No Evidence that Studies Are Biased Due to Self-Selection. Dignity: J Sex Exploit Violence. 2018;3. 10.23860/dignity.2018.03.01.07.

[41] Federal Statistical Office. Total students and German students in the field of medicine (general medicine) by gender. (Statistisches Bundesamt. Studierende insgesamt und Studierende Deutsche im Studienfach Medizin (Allgemein-Medizin) nach Geschlecht.). https://www.destatis.de/DE/Themen/Gesellschaft-Umwelt/Bildung-Forschung-Kultur/Hochschulen/Tabellen/lrbil05.html. Accessed 2 Jan 2025.

