# Correction: Sexual harassment at German medical schools – a national cross-sectional study

**DOI:** 10.1186/s12909-026-10287-7

**Published:** 2026-08-29

**Authors:** Michelle Förstel, Maximilian Vogt, Sabine Drossard

**Affiliations:** 1 Department of Gynecology and Obstetrics, University Hospital Heidelberg, Heidelberg, Germany; 2 German Medical Students’ Association (Bundesvertretung der Medizinstudierenden in Deutschland, bvmd e.V.), Berlin, Germany; 3 Department of Neurosurgery, University Medical Center Greifswald, Greifswald, Germany; 4 Department of General, Visceral, Transplant, Vascular and Pediatric Surgery, University Hospital Würzburg, Würzburg, Germany

**Correction: BMC Medical Education 26, 558 (2026)**

**https://doi.org/10.1186/s12909-026-08890-9**

Following publication of the original article [1], authors would like to update “Among affected students, only 21.6% reported an incident.” into “Among affected students, only 12.7% reported an incident (*n* = 2392).”

The original article [1] has been corrected.
