# Supplementary figures and images for: Sexual harassment at German medical schools – a national cross-sectional study
Source: BMC Med Educ. 2026 Feb 27;26:558. doi: 10.1186/s12909-026-08890-9 (PMC13049711; doi:10.1186/s12909-026-08890-9)

# Supplementary Material

## Appendix 1: Distribution of feedback per faculty

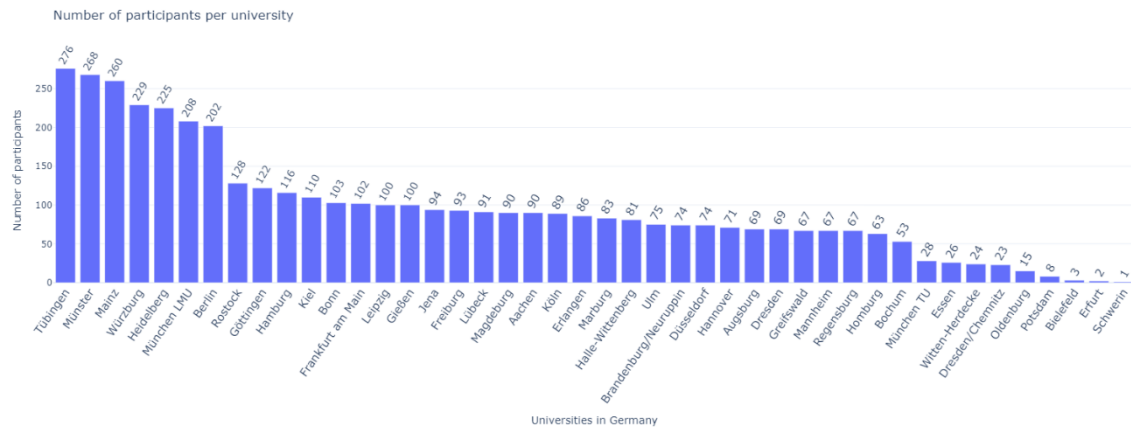

Supplement: Supplementary file 1 — Supplementary Material 1. Appendix 1: Distribution of feedback per faculty. [file 12909_2026_8890_MOESM1_ESM.pdf]
