# Supplementary material for: Sexual harassment at German medical schools – a national cross-sectional study
Source: BMC Med Educ. 2026 Feb 27;26:558. doi: 10.1186/s12909-026-08890-9 (PMC13049711; doi:10.1186/s12909-026-08890-9)
Supplement: Supplementary file 2 — Supplementary Material 2. Appendix 2: PDF of the original questionnaire (in German). [file 12909_2026_8890_MOESM2_ESM.pdf]

#MedToo - sexuelle Belästigung im Medizinstudium

Liebe Studierende,

wir heißen euch herzlich willkommen zur Teilnahme an unserer **Umfrage zur sexuellen Belästigung im Rahmen des Medizinstudiums in Deutschland**. Mit dieser Umfrage streben wir an, ein umfassenderes Verständnis über die Ausmaße und die Auswirkungen von sexueller Belästigung in unserem Studium zu erlangen. Eure Erfahrungen und Beobachtungen sind von unschätzbarem Wert, um ein klares Bild der aktuellen Situation zu erhalten und die Umsetzung möglicher Lösungsansätze stärker in den Mittelpunkt zu rücken.

In dieser Umfrage werden die Häufigkeit, die Umstände und die Auswirkungen von sexueller Belästigung im Studium der Humanmedizin erfragt. Wir bitten euch **nicht** um Angaben zu konkreten Ereignissen und Abläufen. **Die Umfrage ist <u>weitgehend anonym</u>,** das heißt das aufgrund der Datenzusammensetzung Rückschlüsse auf konkrete Personen gezogen werden könnten. Die von euch bereitgestellten personenbezogenen Informationen werden **streng vertraulich behandelt**.

Bitte beachtet: Diese Umfrage enthält Fragen und Themen zu sexueller Belästigung, die belastend sein können. Falls ihr euch durch die Inhalte des Fragebogens überfordert fühlt, sind für euch unter folgendem Link Informationen und Anlaufstellen gesammelt: Antidiskriminierungsstelle des Bundes (https://www.antidiskriminierungsstelle.de/DE/startseite/startseite-node.html) Weiterhin könnt ihr die Umfrage auch pausieren.

Wir bedanken uns für euer Interesse und Engagement. Eure Teilnahme trägt maßgeblich dazu bei auf dieses wichtige Thema aufmerksam zu machen und eine sichere und respektvolle Lernumgebung für alle zu fördern.

Darüber hinaus möchten wir euch bitten, diese Umfrage an eure Kommiliton*innen **weiterzuleiten**, um ein breites Spektrum von Meinungen und Erfahrungen zu sammeln. Gemeinsam können wir einen bedeutenden Schritt zur Verbesserung unseres akademischen Umfelds machen. Die Umfrage wird **zwischen 5 und 20 Minuten** eurer Zeit in Anspruch nehmen (***Mediane Bearbeitungszeit 6:12 min***).

Vielen Dank für eure Zeit!

Michelle Förstel (bvmd), Naila Khan (bvmd), Dr. Sabine Drossard (Uniklink Würzburg), Maximilian Vogt (TU Dresden)

In dieser Umfrage sind 23 Fragen enthalten.

Sexuelle Belästigung im Studium

Das Allgemeine Gleichbehandlungsgesetz (AGG) in Deutschland spricht von einer sexuellen Belästigung, wenn eine **unerwünschte Verhaltensweise**, die sexualisiert oder geschlechtsbezogen ist, **bezweckt oder bewirkt**, **die Würde einer anderen Person zu verletzen**. Das heißt: Das Verhalten **beleidigt**, **erniedrigt** oder **beschämt** die andere Person. Dies kann absichtlich oder unabsichtlich erfolgen. Sexuelle Belästigung ist unter bestimmten Voraussetzungen strafbar. Doch auch **Verhaltensweisen**, die (noch) **nicht strafbar sind**, können **als unangemessen, grenzüberschreitend, respektlos, unangenehm oder herabwürdigend <u>empfunden</u> werden**. Jeder Mensch hat eine individuelle Grenze, wann ein Verhalten eine Verletzung der Würde darstellt und wann nicht. Diese Grenze gilt es bei allen zu respektieren.
Im Rahmen des Medizinstudiums könnten Beispiele für sexuelle Belästigung sein:

- Kommentare über körperliche Merkmale (diese können anzüglich, herablassend oder „nett gemeint“ sein)

- Unangemessene Fragen oder Aussagen zum Privat- und Liebesleben
- Anzügliche Kommentare oder Aufforderungen zu Berührung
- Unaufgefordertes Erzählen von intimen Inhalten
- Witze mit sexistischem und sexuellem Inhalt
- Kommentare über das Geschlecht einer Person
- Anstarren
- Absichtliche oder „scheinbar zufällige" Berührungen
- Einladungen zu privaten Treffen außerhalb der Arbeit
- Unaufgefordertes Zeigen oder Zusenden von sexuellen oder pornographischen Inhalten
- und vieles mehr. Diese Liste hat keinen Anspruch auf Vollständigkeit.

Vgl Allgemeines Gleichbehandlungsgesetz §3

https://www.gesetze-im-internet.de/agg/__3.html (https://www.gesetze-im-internet.de/agg/__3.html)

Warst/Bist du während deines Studiums von sexueller Belästigung betroffen? *

Bitte wählen Sie nur eine der folgenden Antworten aus:

◯ Ja
◯ Nein
◯ Ich bin mir nicht sicher

Hast du während deines Studiums sexuelle Belästigung beobachtet / beobachtest diese?

*

Bitte wählen Sie nur eine der folgenden Antworten aus:

◯ Ja
◯ Nein
◯ Ich bin mir nicht sicher

Sexuelle Belästigung im Studium

Wie oben beschrieben, kann sexuelle Belästigung verschiedene Formen annehmen. Im Folgenden geht es darum, welche Arten von sexueller Belästigung du in welchem Ausmaß erlebt hast.

Hinweis: Du füllst die Umfrage am Handy aus? Im Querformat lassen sich die Tabellen besser ausfüllen!

Wie häufig hast du folgende Arten von sexueller Belästigung im Medizinstudium erlebt oder beobachtet?
*

Bitte wählen Sie die zutreffende Antwort für jeden Punkt aus:

	nie	selten	gelegentlich	oft	sehr oft	ich bin mir nicht sicher
**verbal** unerwünschte, sexuell konnotierte verbale Äußerung	○	○	○	○	○	○
**nonverbal** unerwünschte, sexuell geprägte Handlungen, Blicke oder Gesten	○	○	○	○	○	○
**physisch** unerwünschte körperliche Berührungen oder Annäherungen	○	○	○	○	○	○
**digital** unerwünschte Verhaltensweisen mit sexuellem Bezug über elektronische Kommunikationsmittel	○	○	○	○	○	○

Häufigkeit von sexueller Belästigung

Sexuelle Belästigung kann in verschiedenen Situationen auftreten. Im Folgenden geht es darum, in welchem Kontext und in welchem Ausmaß du sexueller Belästigung ausgesetzt warst.

Hinweis: Du füllst die Umfrage am Handy aus? Im Querformat lassen sich die Tabellen besser ausfüllen!

Wie häufig hast du in den jeweiligen Studienabschnitten sexuelle Belästigung erlebt oder beobachtet?

Ich habe sexuelle Belästigung im … erlebt.

*

Bitte wählen Sie die zutreffende Antwort für jeden Punkt aus:

	(noch) nicht begonnen	nie	selten	gelegentlich	oft	sehr oft	ich bin mir nicht sicher
**Pflegepraktikum**	◯	◯	◯	◯	◯	◯	◯
**Famulatur**	◯	◯	◯	◯	◯	◯	◯
**PJ**	◯	◯	◯	◯	◯	◯	◯
**Theoretischer Unterricht**	◯	◯	◯	◯	◯	◯	◯
**Praktischer Unterricht mit Patient*innen**	◯	◯	◯	◯	◯	◯	◯
**Praktischer Unterricht ohne Patient*innen**	◯	◯	◯	◯	◯	◯	◯
**Im Rahmen der Promotion**	◯	◯	◯	◯	◯	◯	◯
**In anderen studiumsbezogenen Veranstaltungen (Fachschaftsrats-Partys, Erstie-Woche,...)**	◯	◯	◯	◯	◯	◯	◯

Personen von denen sexuelle Belästigung ausgeht

Sexuelle Belästigung kann von verschiedenen Personen ausgehen. Im Folgenden interessieren wir uns dafür, durch welche Personengruppen sexuelle Belästigung ausging.

Hinweis: Du füllst die Umfrage am Handy aus? Im Querformat lassen sich die Tabellen besser ausfüllen!

Wie häufig ging die sexuelle Belästigung von den folgenden Personengruppen in Klinik/Praxis aus? *

Bitte wählen Sie die zutreffende Antwort für jeden Punkt aus:

	nie	selten	gelegentlich	oft	sehr oft	keine Angabe
**Chefärzt*innen /leitende Oberärzt*innen**	○	○	○	○	○	○
**Oberärzt*innen /Fachärzt*innen**	○	○	○	○	○	○
**Assistenzärzt*innen**	○	○	○	○	○	○
**Patient*innen**	○	○	○	○	○	○
**Angehörige**	○	○	○	○	○	○
**Pflegepersonal / MFAs / OTAs**	○	○	○	○	○	○
**andere Berufsgruppen, wie (Physio-, Ergo-, Küchen- oder Reinigungspersonal)**	○	○	○	○	○	○

Wie häufig ging die sexuelle Belästigung von den folgenden Personengruppen an der Universität aus? *

Bitte wählen Sie die zutreffende Antwort für jeden Punkt aus:

	nie	selten	gelegentlich	oft	sehr oft	keine Angabe
**Professor*innen**	○	○	○	○	○	○
**Wiss. Mitarbeiter*innen**	○	○	○	○	○	○
**Kommiliton*innen**	○	○	○	○	○	○
**studentische Tutor*innen**	○	○	○	○	○	○
**nichtwissenschaftliche Mitarbeitende (z.B. Verwaltung, Sicherheitsdienst, Mensapersonal)**	○	○	○	○	○	○

Orte und Fachbereiche mit sexueller Belästigung

Im Folgenden soll es um verschiedene Fachbereiche gehen, an welchen du sexuelle Belästigung erlebt hast.

Wie häufig warst/bist du in deinem Studium im Durchschnitt sexueller Belästigung ausgesetzt?

*

Bitte wählen Sie nur eine der folgenden Antworten aus:

- ◯ Nie
- ◯ Einmalig
- ◯ Ca. 1-3 x pro Jahr
- ◯ Ca. 1-3 x pro Quartal
- ◯ Ca. 1-3 x pro Monat
- ◯ Ca. 1-3 x pro Woche
- ◯ (Beinahe) täglich
- ◯ Mehrmals täglich

An welchem dieser Orte warst / bist du sexueller Belästigung ausgesetzt? (Mehrfachauswahl möglich, **bei nicht zutreffend bitte freilassen**)

Bitte wählen Sie alle zutreffenden Antworten aus:

- ☐ Universitätsklinikum
- ☐ Lehrkrankenhaus
- ☐ Nicht-Lehrkrankenhaus
- ☐ Praxis
- ☐ Universitätsgebäude (Bibliothek, Hörsäale...)

Wie viel Prozent der Personen, von denen sexuelle Belästigung ausging, waren männlich gelesen (in %) ?

❶ Jede Antwort muss zwischen 0 und 100 sein

Bitte geben Sie Ihre Antwort(en) hier ein:

***Weder durch das Aussehen noch den Namen lässt sich das Geschlecht einer Person verlässlich zuordnen.**

In welchem der folgenden Fachbereiche hast du sexuelle Belästigung **erfahren oder beobachtet**? (Mehrfachauswahl möglich, **bei nicht zutreffend bitte freilassen**)

Bitte wählen Sie alle zutreffenden Antworten aus:

- [ ] Allgemeinmedizin
- [ ] Anästhesiologie
- [ ] Augenheilkunde
- [ ] Chirurgie
- [ ] Dermatologie
- [ ] Gynäkologie & Geburtshilfe
- [ ] Innere Medizin
- [ ] HNO
- [ ] Neurologie
- [ ] Pädiatrie
- [ ] Psychiatrie
- [ ] Radiologie
- [ ] Urologie
- [ ] Sonstiges: ____________

In welchem der folgenden chirurgischen Fachbereiche hast du sexuelle Belästigung **erfahren oder beobachtet**? (Mehrfachauswahl möglich) *

Beantworten Sie diese Frage nur, wenn folgende Bedingungen erfüllt sind:
Antwort war bei Frage '10 [Fachbereich]' (In welchem der folgenden Fachbereiche hast du sexuelle Belästigung erfahren oder beobachtet? (Mehrfachauswahl möglich, bei nicht zutreffend bitte freilassen))

Bitte wählen Sie alle zutreffenden Antworten aus:

- ☐ Allgemein- & Viszeralchirurgie
- ☐ Gefäßchirurgie
- ☐ Herz- & Thoraxchirurgie
- ☐ Kinderchirurgie
- ☐ Mund-Kiefer-Gesichtschirurgie
- ☐ Neurochirurgie
- ☐ Unfallchirurgie & Orthopädie
- ☐ nicht näher spezifizierbar/ keine Angabe
- ☐ Sonstiges: ____________

Auswirkung der sexuellen Belästigung

Wir wollen ein besseres Verständnis dafür entwickeln, wie sich sexuelle Belästigung auf das Wohlbefinden und die akademische Leistung der Studierenden auswirkt. Im Folgenden soll es daher um die Auswirkungen von sexueller Belästigung gehen.

Wie sehr fühlst du dich von sexueller Belästigung im Rahmen des Studiums belastet?

❗ Jede Antwort muss zwischen 0 und 10 sein
Bitte geben Sie Ihre Antwort(en) hier ein:

Auf einer Skala von

Inwiefern hat sexuelle Belästigung dein Studium oder deine Karriere beeinflusst? (Mehrfachauswahl möglich)

(Im Folgenden nur eine Auswahl)

Bitte wählen Sie alle zutreffenden Antworten aus:

- ☐ Sexuelle Belästigung hat **keinen** negativen Einfluss auf mein Studium und meine Karriere.
- ☐ Meine **Noten** haben sich verschlechtert.
- ☐ Ich habe darüber nachgedacht, das Studium **abzubrechen**.
- ☐ Ich bin **unzufrieden** mit meinem Studium.
- ☐ Es hat Einfluss auf meine **Wahl** von Praktikumsplätzen/Famulaturenn/PJ-Wahltertialen.
- ☐ Ich habe erwogen, meine **Promotion** abzubrechen oder gar nicht erst dort zu beginnen.
- ☐ Es hat Einfluss auf die Wahl meiner fachärztlichen **Weiterbildung**.
- ☐ Es hat Einfluss auf die Wahl meines zukünftigen **Arbeitsplatzes**
- ☐ Es hat Auswirkungen auf mein (psychisches) **Wohlbefinden** (z.B. Stress, Angst).
- ☐ Ich **vermeide** spezifische Situationen oder Personen.

Lösungsansätze und Anlaufstellen

Im Folgenden soll es um deine Erfahrungen mit Meldestellen im Kontext sexueller Belästigung gehen.

Bitte bewerte die folgenden Aussagen: **An meiner Universität/ meiner medizinischen Fakultät... ***

Bitte wählen Sie die zutreffende Antwort für jeden Punkt aus:

	stimme ganz und gar nicht zu	stimme eher nicht zu	teils/teils	stimme eher zu	stimme voll und ganz zu	keine Aussage möglich
**...kenne Ich die Stellen, an die ich mich bei sexueller Belästigung wenden kann.**	○	○	○	○	○	○
**...gibt es ausreichend Angebote zur Meldung von sexueller Belästigung.**	○	○	○	○	○	○
**...werden ausreichend Möglichkeiten zur psychosozialen Unterstützung bei sexueller Belästigung angeboten.**	○	○	○	○	○	○
**...gibt es klare Richtlinien (Handlungsanweisungen) zum Umgang mit sexueller Belästigung.**	○	○	○	○	○	○
**...wurde in der Vergangenheit ein konsequentes Durchgreifen bei Fällen von sexueller Belästigung gezeigt.**	○	○	○	○	○	○
**...empfinde Ich die Angebote zur Beratung und Meldung bei sexueller Belästigung, als niederschwellig.**	○	○	○	○	○	○

	stimme ganz und gar nicht zu	stimme eher nicht zu	teils/teils	stimme eher zu	stimme voll und ganz zu	keine Aussage möglich
**... wird für das Thema sexuelle Belästigung proaktiv aufgeklärt und sensibilisiert.**	◯	◯	◯	◯	◯	◯

Hast du bereits einen Vorfall von sexueller Belästigung gemeldet?

Damit sind vor allem auf lokaler Ebene Hilfe-Strukturen gemeint - z.B. Vertrauenspersonen, Beschwerdestellen, Gleichstellungsbeauftragte.

*

Beantworten Sie diese Frage nur, wenn folgende Bedingungen erfüllt sind:
((SexBe.NAOK (/umfragen/limesurvey/index.php/admin/questions/sa/view/surveyid/868571/gid/108870/qid/1194332) == "Ja") or (SexBeObserv.NAOK (/umfragen/limesurvey/index.php/admin/questions/sa/view/surveyid/868571/gid/108870/qid/1267295) == "A1"))

Bitte wählen Sie nur eine der folgenden Antworten aus:

◯ Ja

◯ Nein

◯ Keine Angabe

Bitte bewerte die folgenden Aussagen: *

Beantworten Sie diese Frage nur, wenn folgende Bedingungen erfüllt sind:
(((SexBe.NAOK
(/umfragen/limesurvey/index.php/admin/questions/sa/view/surveyid/868571/gid/108870/qid/1194332)
== "Ja") or (SexBeObserv.NAOK
(/umfragen/limesurvey/index.php/admin/questions/sa/view/surveyid/868571/gid/108870/qid/1267295)
== "A1")) and (Gemeldet.NAOK
(/umfragen/limesurvey/index.php/admin/questions/sa/view/surveyid/868571/gid/100788/qid/1194336)
== "A1"))

Bitte wählen Sie die zutreffende Antwort für jeden Punkt aus:

	**stimme ganz und gar nicht zu**	**stimme eher nicht zu**	**teils/teils**	**stimme eher zu**	**stimme voll und ganz zu**	**keine Aussage möglich**
**Ich bin zufrieden mit dem Meldeprozess.**	○	○	○	○	○	○
**Ich habe ausreichend psychosoziale Unterstützung erhalten.**	○	○	○	○	○	○
**Ich habe ausreichend rechtliche Beratung erhalten.**	○	○	○	○	○	○
**Ich habe den Meldeprozess als belastend empfunden.**	○	○	○	○	○	○
**Ich bin zufrieden mit der Konsequenz, die meine Meldung hatte.**	○	○	○	○	○	○
**Ich würde mich bei einem weiteren Vorfall wieder für eine Meldung entscheiden.**	○	○	○	○	○	○

Was hat dich davon abgehalten, einen Vorfall sexueller Belästigung zu melden? (Mehrfachnennung möglich) *

Beantworten Sie diese Frage nur, wenn folgende Bedingungen erfüllt sind:
(((SexBe.NAOK (/umfragen/limesurvey/index.php/admin/questions/sa/view/surveyid/868571/gid/108870/qid/1194332) == "Ja") or (SexBeObserv.NAOK (/umfragen/limesurvey/index.php/admin/questions/sa/view/surveyid/868571/gid/108870/qid/1267295) == "A1")) and (Gemeldet.NAOK (/umfragen/limesurvey/index.php/admin/questions/sa/view/surveyid/868571/gid/100788/qid/1194336) == "A2"))

Bitte wählen Sie alle zutreffenden Antworten aus:

☐

Struktur/Prozess

Dazu könnte zum Beispiel folgendes gehören:

- Es gab keine Meldestelle
- Ich weiß nicht, wer dafür im konkreten Fall zuständig gewesen wäre
- Die Meldemöglichkeit war nicht attraktiv
- Es hat sich niemand zuständig gefühlt

☐

Emotionen

Dazu könnte zum Beispiel folgendes gehören:

- Ich wollte mich nicht damit auseinandersetzen
- Der Patient, von dem die Belästigung ausging, tat mir leid.
- Ich habe mich geschähmt

☐

Karriere/Folgen

Dazu könnte zum Beispiel folgendes gehören:

- Potenzielle negative Folgen für mich
- Ich wollte nicht "negativ" auffallen

☐

Unsicherheit bei der Einordnung

Dazu könnte zum Beispiel folgendes gehören:

- Ich war mir nicht sicher, ob es sich um sexuelle Belästigung gehandelt hat.
- Ich habe mich an die Situation nicht gut genug erinnert
- Ich habe das Handeln als “normal” im Kontext/Berufsalltag der Situation eingeordnet
- Mir wurde gesagt, dass wäre normal / Ich solle mich nicht so anstellen

☐

Mangelnde Konsequenz

Dazu könnte zum Beispiel folgendes gehören:

- Ich habe nicht gedacht, dass es etwas bringt
- Mir wurde gesagt "das bringe doch eh nichts"
- Ich habe schonmal erlebt, dass dadurch nichts passiert ist.

Persönliche Angaben

Welchem Geschlecht ordnest du dich zu? *

Bitte wählen Sie nur eine der folgenden Antworten aus:

- ◯ weiblich
- ◯ männlich
- ◯ andere (wie non binär)
- ◯ keine Angabe

Wie alt bist du? *

Bitte wählen Sie nur eine der folgenden Antworten aus:

- ◯ ≤ 19
- ◯ 20-24
- ◯ 25-29
- ◯ 30-34
- ◯ ≥ 35
- ◯ keine Angabe

Hast du vor dem Medizinstudium bereits gearbeitet oder eine Ausbildung / ein Studium absolviert? *

Bitte wählen Sie nur eine der folgenden Antworten aus:

- ◯ Nein
- ◯ Ja, Ausbildung im medizinnahen Bereich (z.B. Gesundheits- & Krankenpflege, Rettungsdienst, Physiotherapie)
- ◯ Ja, sonstige Tätigkeit im medizinnahen Bereich (z.B. FSJ, BFD)
- ◯ Ja, im nicht-medizinischen Bereich
- ◯ Keine Angabe
- ◯ Sonstiges ____________

An welchem Standort studierst du aktuell? *

Bitte wählen Sie nur eine der folgenden Antworten aus:

- ◯ Aachen
- ◯ Augsburg
- ◯ Berlin
- ◯ Bielefeld
- ◯ Bochum
- ◯ Bonn
- ◯ Brandenburg/Neuruppin
- ◯ Dresden/Chemnitz
- ◯ Dresden
- ◯ Düsseldorf
- ◯ Erlangen
- ◯ Essen
- ◯ Frankfurt am Main
- ◯ Freiburg
- ◯ Gießen
- ◯ Göttingen
- ◯ Greifswald
- ◯ Halle-Wittenberg
- ◯ Hamburg
- ◯ Hannover
- ◯ Heidelberg
- ◯ Homburg
- ◯ Jena
- ◯ Kiel
- ◯ Köln
- ◯ Leipzig
- ◯ Lübeck
- ◯ Magdeburg
- ◯ Mainz
- ◯ Mannheim
- ◯ Marburg

◯ München LMU

◯ München TU

◯ Münster

◯ Oldenburg

◯ Regensburg

◯ Rostock

◯ Saarland

◯ Tübingen

◯ Ulm

◯ Witten-Herdecke

◯ Würzburg

◯ keine Angabe

◯ Sonstiges

Hast du Teile deines Studiums an einer anderen Universität absolviert? *

Bitte wählen Sie nur eine der folgenden Antworten aus:

◯ Ja, an einer anderen Fakultät in Deutschland

◯ Ja, an einer anderen Fakultät außerhalb Deutschlands (zum Beispiel ERASMUS, oder Vorklinik im Ausland)

◯ Nein

In welchem Studienabschnitt befindest du dich aktuell? *

Bitte wählen Sie nur eine der folgenden Antworten aus:

◯ Vorklinik (vor M1 bzw. Äquivalent)

◯ Klinik (zwischen M1 und M2)

◯ Praktisches Jahr (zwischen M2 und M3)

◯ Keine Angabe

Wir möchten uns herzlich bei dir für deine Hilfe bedanken. Dein Mitwirken ist ein wertvoller Beitrag, um mehr Informationen zu diesem Thema zu erfassen und somit auch mehr Aufmerksamkeit zu erhalten**.**

Vielen Dank nochmals für deine Zeit und Engagement!

Teile gerne die Umfrage an deine Kommililton*innen!
Bei Fragen/Feedback zur Umfrage kannst du dich jederzeit an medtoo@bvmd.de (mailto:medtoo@bvmd.de) wenden.

Solltest du Unterstützung benötigen und nicht wissen, an wen du dich wenden kannst, kannst du ber folgende Website auf weitere Informationen und Unterstützungsangebote zugreifen: Antidiskriminierungsstelle des Bundes (https://www.antidiskriminierungsstelle.de/DE/startseite/startseite-node.html)

01.10.2024 – 12:59

Übermittlung Ihres ausgefüllten Fragebogens:
Vielen Dank für die Beantwortung des Fragebogens.
