# Supplementary material for: Sexual harassment at German medical schools – a national cross-sectional study
Source: BMC Med Educ. 2026 Feb 27;26:558. doi: 10.1186/s12909-026-08890-9 (PMC13049711; doi:10.1186/s12909-026-08890-9)
Supplement: Supplementary file 3 — Supplementary Material 3. Appendix 3: PDF of translated questionnaire. [file 12909_2026_8890_MOESM3_ESM.pdf]

#MedToo - sexual harassment in medical studies

Dear students,

We would like to invite you to participate in our survey on sexual harassment during medical studies in Germany. With this survey we aim to gain a more comprehensive understanding of the forms and effects of sexual harassment in our studies. Your experiences and observations are invaluable in gaining a clear picture of the current situation and focusing more on the implementation of possible solutions.

This survey asks about the frequency, circumstances and impact of sexual harassment in medical school. We do not ask for information on specific events or processes. The survey is largely anonymous and the personal information you provide will be kept strictly confidential.

Please note: This survey contains questions and topics about sexual harassment that may be distressing. If you feel distressed by the contents of the questionnaire, we have provided information and contact points under the following link: ...

Thank you for your interest and commitment. Your participation will make a significant contribution to raising awareness of this important issue and creating a safer and more respectful learning environment for all.

In addition, we would like to ask you to forward this survey to your fellow students in order to gather a wide range of opinions and experiences. Together we can take a significant step towards improving our academic environment. The survey will take between 5 and 25 minutes of your time (median time: 6:12 min).

Thank you for your time!

There are 24 questions in this survey.

Sexual harassment during studies

The General Equal Treatment Act (AGG) in Germany speaks of sexual harassment when unwanted behavior that is sexualized or gender-related has the purpose or effect of violating the dignity of another person. This means that the behaviour insults, humiliates or shames the other person.

This can be done intentionally or unintentionally. Sexual harassment is punishable under certain circumstances. However, behavior that is not (yet) punishable can also be perceived as inappropriate, transgressive, disrespectful, unpleasant or degrading. Every person has an individual limit as to when behavior constitutes a violation of dignity and when it does not. This boundary must be respected by everyone.

In the context of medical studies, examples of sexual harassment could include

Comments about physical features (these can be suggestive, patronizing or ‘nice‘)

Inappropriate questions or statements about private and love life

Suggestive comments or requests for touching

Unsolicited sharing of intimate content

Jokes with sexist and sexual content

Comments about a person's gender

Staring

Intentional or ‘seemingly accidental’ touching

Invitations to private meetings outside of work

Unsolicited showing or sending of sexual or pornographic content

and much more. This list is not exhaustive. See General Equal Treatment Act §3

https://www.gesetze-im-internet.de/agg/__3.html

Have you been/are you affected by sexual harassment during your studies?

Yes

No

I am not sure

Did you observe / do you observe sexual harassment during your studies?

Yes

No

I am not sure

Sexual harassment at university

As described above, sexual harassment can take various forms. The following is about what types of sexual harassment you have experienced and to what extent.

How often have you experienced or observed the following types of sexual harassment in medical school?

	never	rarely	occasionally	often	very often	I'm not sure
**verbal**<br>unwanted, sexually connoted verbal expression						
**non-verbal**<br>unwanted, sexualized actions, looks or gestures						
**physically**<br>unwanted physical touching or advances						
**digital**<br>unwanted behavior with a sexual connotation via electronic means of communication						

Frequency of sexual harassment

Sexual harassment can occur in various situations. The following is about the context and extent to which you have been exposed to sexual harassment.

How often did you experience or observe sexual harassment in the respective study periods?

I have experienced sexual harassment in ...

	Not (yet) started	never	rarely	occasionally	often	very often	I'm not sure
Nursing internship							
Clinical clerkship							
Practical year							
Theoretical teaching							
Practical lessons with patients							
Practical lessons without patients							
in the context of the doctoral thesis							
In other study-related events (student council parties, fresher's week,...)							

Perpetrators

Sexual harassment can be perpetrated by various people. In the following, we are interested in which groups of people have caused sexual harassment.

How frequently did you experience sexual harassment from the following groups of people in the clinic/practice?

	never	rarely	occasionally	often	very often	not specified
Chief physicians / Senior consultants						
Senior physicians / Attendings						
Residents						
Patients						
Relatives						
Nursing staff / MFAs / OTAs						
Other professional groups, (such as physiotherapy, occupational therapy, kitchen or cleaning staff)						

How frequently did you experience sexual harassment from the following groups of people at the university?

	never	rarely	occasionally	often	very often	not specified
Professors						
Academic staff						
fellow students						
Student tutors						
Non-academic staff (e.g. administration, security, cafeteria staff)						

Places and departments with sexual harassment

The following is about different departments where you have experienced sexual harassment.

On average, how often were/are you exposed to sexual harassment during your studies?
Never
Once
Approx. 1-3 x per year
Approx. 1-3 x per quarter
Approx. 1-3 x per month
Approx. 1-3 x per week
(Almost) daily
Several times a day

In which of these places were/are you exposed to sexual harassment? (Multiple choice possible, please leave blank if not applicable)
University hospital
Teaching hospital
Non-teaching hospital
Medical practice
University building (library, auditoriums ...)

What percentage of people who were the source of sexual harassment were male (in %)?

0-100

In which of the following subject areas have you experienced or observed sexual harassment? (multiple choice possible, please leave blank if not applicable)
General medicine
Anesthesiology
Ophthalmology
Surgery
Dermatology
Gynecology & Obstetrics
Internal Medicine
ENT
Neurology
Pediatrics
Psychiatry
Radiology
Urology
Other:

In which of the following surgical specialties have you experienced or observed sexual harassment? (multiple choice possible) * (only when Surgery was chosen)

General & Visceral Surgery
Vascular Surgery
Cardiac & Thoracic Surgery
Pediatric Surgery
Oral and Maxillofacial Surgery
Neurosurgery
Trauma Surgery & Orthopedics
Not specified
Other:

Consequences of sexual harassment

We want to develop a better understanding of how sexual harassment affects the well-being and academic performance of students. The following will therefore focus on the impact of sexual harassment.

How much do you feel affected by sexual harassment during your studies?

On a scale of 1 to 10

To what extent has sexual harassment affected your studies or career? (multiple choice possible)

Sexual harassment has had no negative impact on my studies or career.

My grades have deteriorated.

I have thought about dropping out of my studies.

I am dissatisfied with my studies.

It has influenced my choice of internships/traineeships/PJ electives.

I have considered cancelling my doctorate or not starting it at all.

It has influenced my choice of specialist medical training.

It has an influence on my choice of future job

It has an impact on my (mental) well-being (e.g. stress, anxiety).

I avoid specific situations or people

Solutions and contact points

The following is about your experiences with reporting offices in the context of sexual harassment.

Please rate the following statements: At my university/my medical faculty... *
...I know the offices I can contact in the event of sexual harassment.
...there are sufficient opportunities to report sexual harassment.
...there are sufficient opportunities for psychosocial support in the event of sexual harassment.
...there are clear guidelines (instructions for action) for dealing with sexual harassment.
...there has been a consistent response to cases of sexual harassment in the past.
...I feel that the counselling and reporting services for sexual harassment are low-threshold.
... is proactively educated and sensitized to the issue of sexual harassment.
Scale:
Strongly disagree
Disagree
Neither agree nor disagree
Agree
Fully Agree
Prefer not to answer

Have you already reported an incident of sexual harassment?
Yes
No
Not specified

Only if yes: Please rate the following statements:

I am satisfied with the reporting process.
I have received sufficient psychosocial support.
I have received sufficient legal counselling.
I found the reporting process stressful.
I am satisfied with the consequences of my report.
I would decide to report again if there was another incident.

Scale:
Strongly disagree
Disagree
Neither agree nor disagree
Agree
Fully Agree
Prefer not to answer

What prevented you from reporting an incident of sexual harassment? (multiple answers possible) *

Structure/Process

This could include, for example, the following:

- There was no reporting office
- I do not know who would have been responsible in the specific case
- The reporting option was not attractive
- Nobody felt responsible

Emotions

- I didn't want to deal with it
- I felt sorry for the patient who was the source of the harassment.
- I felt ashamed

Career/Consequences

- Potential negative consequences for me
- I didn't want to attract 'negative' attention
- Uncertainty about categorization
- This could include the following, for example:

Uncertainty in Categorization

- I wasn't sure if it was sexual harassment.
- I didn't remember the situation well enough
- I categorized the actions as 'normal' in the context/work environment of the situation
- I was told that it was normal / I shouldn't behave like that

Lack of Consistency

- I didn't think it would do any good
- I was told 'it won't help anyway'
- I have already experienced that nothing happened as a result.

Personal details

What is your self-identified gender?
Female
male
other (e.g. non binary)
Prefer not to say

How old are you?
≤ 19
20-24
25-29
30-34
≥ 35
Prefer not to say

Did you already work or complete an apprenticeship/study program before studying medicine?
No
Yes, training in a medical-related field (e.g. health & nursing, emergency services, physiotherapy)
Yes, other work in the medical field (e.g. FSJ, BFD)
Yes, in a non-medical field
Prefer not to say
Something else:

Where are you currently studying? *
Aachen
Augsburg, Berlin, Bielefeld, Bochum, Bonn, Brandenburg/Neuruppin, Dresden/Chemnitz, Dresden, Düsseldorf, Erlangen, Essen, Frankfurt am Main, Freiburg, Gießen, Göttingen, Greifswald, Halle-Wittenberg, Hamburg, Hannover, Heidelberg, Homburg, Jena, Kiel, Köln, Leipzig, Lübeck, Magdeburg, Mainz, Mannheim, Marburg, München LMU, München TU, Münster, Oldenburg, Regensburg, Rostock, Saarland, Tübingen, Ulm, Witten-Herdecke, Würzburg, somewhere else: (free text)

Did you complete parts of your degree programme at another university?
Yes, at another faculty in Germany
Yes, at another faculty outside Germany (for example ERASMUS, or pre-clinical training abroad)
No

What stage of your studies are you currently in? *
Pre-clinical Science   Clinical Science   Practical Year   Prefer not to say
